# Perspectives of Phage–Eukaryotic Cell Interactions to Control Epstein–Barr Virus Infections

**DOI:** 10.3389/fmicb.2018.00630

**Published:** 2018-04-03

**Authors:** Andrzej Górski, Ryszard Międzybrodzki, Ewa Jończyk-Matysiak, Beata Weber-Dąbrowska, Natalia Bagińska, Jan Borysowski

**Affiliations:** ^1^Bacteriophage Laboratory, Ludwik Hirszfeld Institute of Immunology and Experimental Therapy, Polish Academy of Sciences, Wrocław, Poland; ^2^Phage Therapy Unit, Ludwik Hirszfeld Institute of Immunology and Experimental Therapy, Polish Academy of Sciences, Wrocław, Poland; ^3^Department of Clinical Immunology, Transplantation Institute, Medical University of Warsaw, Warsaw, Poland

**Keywords:** phage, Epstein–Barr virus, herpes virus, integrin, KGD motif, immunomodulation

## Abstract

Recently, leading medical journals emphasized the importance of further studies on the potential application of bacterial viruses (phages) for the treatment of antibiotics-resistant infections outlining the present status of the therapy and perspectives for the future. Furthermore, a leading scientific journal pointed to the recent progress in research on phage interactions with eukaryotic cells (especially cells of the immune system) and potential implications of their results for our broader understanding of the role of phages – not only as “bacteria eaters” – but also as an important part of our body defense protecting against external and internal pathogenic invaders (as suggested previously). This illustrates how our understanding of the actual role and potential of phages is expanding and how worldwide interest in their use in medicine is growing. In this article we envision how this advancement of our knowledge about phages could be translated into the progress in combating herpesvirus infections especially those caused by Epstein–Barr virus (EBV).

## Introduction

As assessed by serology, EBV infects >90% of the human population; its predominant host cells are B cells and epithelial cells with life-long latency established in the latter cells (Connolly et al., 2011). EBV contributes to 2% of the overall tumor burden, but the majority of infected individuals do not have symptoms of disease. This life-long “peaceful relationship” suggests the involvement of host protective mechanisms (immune system). Our current understanding of the immune response to EBV in healthy and immunocompromised subjects has recently been reviewed (Martinez and Krams, 2017). Infection with EBV may cause infectious mononucleosis and indefinite persistence of the virus in B cells leading to hematopoietic cancer (Burkitt’s lymphoma, Hodgkin’s lymphoma) and lymphoproliferative disorders in patients with immune deficiency (HIV, transplant recipients on immunosuppression). Another clinical setting associated with the virus is chronic active EBV disease (CAEBV), a rare, progressive disorder with infiltration of organs by EBV+ lymphocytes, immunodeficiency, opportunistic infections, and the development of lymphomas (Kimura and Cohen, 2017). Recently, it has been demonstrated that EBV may induce neoplasm development via NF-κB activation not only in B cells but also in T and NK cells (Takada et al., 2017). EBV can also affect epithelial cells and its role has been implicated in the development of nasolaryngeal carcinoma and gastric carcinonoma (EBV may promote chronic inflammation and increased tissue damage) (Morales-Sanchez and Fuentes-Panana, 2017; Pei et al., 2017). Moreover, EBV has been associated with inflammatory bowel disease: the presence of EBV DNA has been identified in 70% of patients with ulcerative colitis but in none with irritable bowel syndrome (Rizzo et al., 2017). It has also been suggested that the well-established association of EBV infection with increased susceptibility to multiple sclerosis is linked to upregulated ability of B cells from patients with the disease to process and present myelin autoantigen; in fact, upregulation of HLA class I and class II molecules has been detected on B cells thus “empowering B cells for autoimmunity” (Morandi et al., 2017). Association of EBV infection has also been suggested in autoimmune syndromes including rheumatoid arthritis (Draborg et al., 2013; Balandraud and Roudier, 2017), systemic lupus erythematosus (Piroozmand et al., 2017) and steroid-sensitive nephrotic syndrome (Dossier et al., 2017). Cytotoxic T cells, NK and NKT cells are believed to contribute to body defenses against EBV, yet our understanding of body defenses against EBV are incomplete. Loss of immunosurveillance predisposes to EBV malignancies; however, such pathology develops in up to 20% of patients receiving immunosuppression after organ transplantation. Why the remaining patients do not develop malignancies is not known, a question of obvious practical significance for the prevention of the development of EBV-dependent complications.

Preclinical and clinical evidence indicates that oxidative stress is an important molecular mechanism in EBV lytic reactivation; it has even been proposed that EBV-induced cancers are ROS-driven (Hu et al., 2017). In addition, there are initial observations showing the preventive value of anti-oxidants in EBV-induced cancer thus suggesting that reactive oxygen species blockade followed by chemotherapy or radiation therapy should offer a more efficient means of EBV-cancer treatment (Huang et al., 2013; Hu et al., 2017). Standard efficient prophylaxis to prevent EBV infection and EBV reactivation is not available; reduction of immunosuppression is the only option while it predisposes to the risk of allograft rejection (Prockop and Vatsayan, 2017). Treatment of EBV-associated diseases includes nucleoside analogs (only affecting EBV lytic cycle). There are indications that some natural components may be active but their mechanism of action remains unclear (Jha et al., 2016). However, it has been demonstrated that apigenin (a natural plant product belonging to the favone group and a strong ROS scavenger) inhibits EBV reactivation (Li et al., 2016). No EBV-targeted therapies are available to control EBV-induced cancers (Fitzsimmons and Kelly, 2017).

The progress in our understanding of clinical and immunopathological aspects of EBV infection is paralleled by a concurrent advancement in our knowledge of phage biology and phage therapy. During the past year at least 10 relevant reviews have been published including commentaries in Lancet (Watts, 2017) and JAMA (Lyon, 2017). Furthermore, data have been accumulating to confirm that the therapy has “potential for evolving from merely a treatment for complications to targeting diseases” (Górski et al., 2016). Accordingly, we postulate that phage therapy may extend beyond its antibacterial action (Górski and Weber-Dąbrowska, 2005) and be applied as an immunomodulatory treatment in inflammatory bowel disease, sepsis autoimmune hepatitis, and allergy (Górski et al., 2017a,b,c, 2018).

## Potential Molecular Basis for Phage–Integrin Interactions

We have put forward a hypothesis of probable molecular basis for T4 phage – mammalian cell interactions based on the presence in the phage head vertex of a protein gp24 containing a KGD (Lys-Gly-Asp) motif known so far to interact with β-3 integrin abundantly present on platelets (Górski et al., 2003). Subsequently, the presence of gene coding for KGD has also been confirmed in T2 phage (Dabrowska et al., 2007). Protein gp24 is highly expressed and located in five copies on each corner of the phage head (55 copies of each phage particle). Its potential interactions are markedly enhanced by this exposure while the functional significance of this motif is confirmed by demonstrated KGD-dependent phage interactions with platelets and blockade of those interactions with a peptide containing the KGD sequence (Integrilin) as well as anti-β3 antibody; what is more, αIIb-β3 integrin-deficient platelets do not interact with phages (Dabrowska et al., 2004a). Furthermore, KGD+ phages can block platelet αIIb-β3 integrin interactions with their major ligand fibrinogen (Kniotek et al., 2004) as well as B16 melanoma cell adhesion to fibrinogen (β3 integrin silencing abolished that phenomenon). Phage–platelets interactions are especially interesting in light of the data pointing to the role of platelets in immune response and inflammation. Recently, it has been shown that platelets can actively migrate (a phenomenon dependent on integrin αIIb-β3 engagement) and collect bacteria forming bacterial biofilm-like structures thus establishing the first line of host defense. Subsequently, neutrophils are recruited that phagocytose platelets–bacteria complexes leading to neutrophil-mediated inflammation blockade of platelets αIIb-β3 abolished platelets migration and subsequent neutrophil activation (Gaertner et al., 2017). Those data point to platelets as potential targets to downregulate inflammation and resulting tissue damage and suggest that phage therapy might be helpful to control that pathology.

Integrin β3 involvement has also been demonstrated in phage-mediated anti-tumor effects in mice (Budynek et al., 2010). This appears to be important in view of the fact that αIIb-β3 integrin can also be ectopically expressed on tumor cells (Timar et al., 1998) and be acquired by other cells (for example, T lymphocytes) as a result of coating by platelet-derived microvesicles containing that integrin (Wierzbicki et al., 2006). “Phage opsonization” of T cells may interfere with their activation and eventually lead to their clearance from the circulation – a phenomenon similar to that occurring using anti-CD3 monoclonal antibody and anti-lymphocyte globulin treatment. KGD is also known to be present within the CD40 ligand (CD40L) which together with CD40 forms an important dyad relevant for mounting an immune response, autoimmunity, and inflammation; for example, its role has recently been suggested in immunopathology of certain forms of glomerulonephritis (Doublier et al., 2017). All those data suggest that the KGD motif present in phages is functional and may mediate interactions of phages with cells of immune system relevant for the development of immune-mediated diseases.

## Phages Against Pathogenic Viruses (PV)?

There are data in the literature which may suggest that phages can mediate anti-PV effects (for review, see Miȩdzybrodzki et al., 2005). Thus, phage-derived nucleic acids may inhibit PV infection by inducing interferons. IFN-alpha and IFN-beta can be induced by short single-stranded RNA transcribed with T3, T7, and Sp6 phage RNA polymerases (Kim et al., 2004). Phage-derived nucleic acids can inhibit *in vitro* HSV infection of kidney cells and *in vivo* genital infections by HSV in guinea pigs. In a model of duck hepatitis B virus M13 phage DNA was even superior to acyclovir. Also, dsRNA from *E. coli* phage protected mice infected with encephalomyocarditis virus. M13 phage DNA as well as whole T4 coliphage were capable of inducing IFN in blood. Increased IFN-gamma production was also observed in mice orally fed with bacteriophage T7 (Park et al., 2014). However, more recent data using purified phage preparations with the lowest achievable endotoxin levels suggest that at least some of those effects could be attributed to residual endotoxin (Dufour et al., 2016), therefore, more studies are needed to determine phage effects on interferon production by cells of the immune system and cells from other tissues (for review, see Miȩdzybrodzki et al., 2005) the alternative mechanism may be based on phage competition with PV for cellular receptors enabling viral infection. In fact, integrins are used as adhesion receptors by some PV and it was shown by Gerlag et al. (2001) that also a filamentous fd phage displaying an RGD peptide could bind to αvβ3 and αvβ5 integrin. Our group has shown that T4 phage inhibits adsorption and replication of human adenovirus *in vitro* in a dose-dependent manner (Przybylski et al., 2015). Although the exact molecular mechanism has not been elucidated, those data suggest that further studies on the phenomenon of phage–PV interference are warranted.

## Phages Against EBV – and Perhaps Other Viral Infections?

The studies of Chesnokova et al. (2009) and Chesnokova and Hutt-Fletcher (2011) have revealed the mechanism of EBV fusion with epithelial cells which is dependent on viral glycoprotein complex gHgL interacting with epithelial integrins ανβ5, ανβ6, or ανβ8. Glycoprotein gHgL binds with high affinity to epithelial cell integrin via prominent KGD motif located on its surface; this was confirmed by ability of KGD-containing peptides to block gHgL binding and EBV infection. Most recent data have confirmed and expanded the role of the KGD motif showing that it is a bifunctional domain mediating EBV fusion of epithelial cells and B cells through interactions with the EBV epithelial integrin receptor or protein gp42. KGD binds to integrin on epithelial cells while B cells are infected through KGD interaction with gp42 – thus KGD “orchestrates EBV infection of both epithelial and B cells” (Chen et al., 2012). Interestingly, glycoproteins gH and gL are conserved across all known human herpesviruses suggesting that their functions in membrane fusion and virus entry are conserved as well. This does not exclude additional role of other non-conserved viral proteins in membrane fusion and entry (Mullen et al., 2002; Sathiyamoorthy et al., 2017a).

The data pointing to an important role of the KGD motif in EBV infection of epithelial cells and B lymphocytes highlight the significance of our findings demonstrating the presence of the same KGD motif in the gp24 head vertex protein of T4-like phages and their potential immunomodulating activity which may take place not only locally but also at other tissue sites via phage translocation from the intestinal tract (Górski et al., 2006). This broader activity of phages has been verified by the recent data confirming our initial assumptions and showing that indeed phages can migrate within epithelial cells (Lehti et al., 2017). As mentioned earlier, KGD+ peptides may inhibit EBV infection. Therefore, KGD+ phages could act in a similar way and prevent EBV infectivity by competitive binding to cellular integrins on epithelial cells and to gp42 protein of the virus itself. Since gHgL is present in all human herpesviruses (Sathiyamoorthy et al., 2017b) it cannot be excluded that such T4 phage-mediated interference with EBV infectivity could occur when KGD+ phages are confronted with other members of the herpesevirus family as well.

It should be noted that phages can also interfere with viral-induced pathology as a consequence of their well-known ability to exert anti- inflammatory action (Górski et al., 2017; Van Belleghem et al., 2017). In this sense, phages could limit EBV-induced inflammatory responses and therefore prevent progression to cancer, which may take place in later stages of EBV infections (Khan, 2006). In fact, disproportionately high EBV DNA levels in inflamed gastrointestinal mucosa suggests that EBV infection may contribute to the pathogenesis of gastritis and inflammatory bowel disease (Ryan et al., 2012). Phages could also exert anti-cancer activity through their anti-NF-κB action (Górski et al., 2017). Furthermore, antioxidant action of phages (Górski et al., 2017) could inhibit EBV lytic reactivation.

The reported interactions of the KGD motif with integrin αvβ6 appear to be of special interest in view of the associations of this receptor with EBV lytic reactivation and tumorigenesis. Upregulation of αvβ6 or αvβ8 integrin increases activation of transforming growth factor – β (TGF-β) which induces lytic reactivation (Chesnokova et al., 2009). The integrin is absent or poorly expressed by normal epithelial cells while it is upregulated in inflammation and cancer. Evidence has accumulated to indicate that the integrin is involved in tumorigenesis and tumor progression: its expression is correlated with a more aggressive cancer and poor patient outcome. Therefore, αvβ6 integrin has recently been a potential target for the development of specific peptidic ligands that could interfere with its activity (Nieberler et al., 2017; Niu et al., 2017). Therefore, KGD+ phages could also interfere with integrin αvβ6-dependent tumorigenesis and tumor progression extending our earlier observations of anti-cancer effects of such phages (phage interference with β3 integrin in a mouse model) (Dabrowska et al., 2004b; Budynek et al., 2010).

It would be of interest to determine if the KGD motif present in the gHgL protein complex may also interact with αIIbβ3 – a platelet integrin reactive with this sequence. EBV infection is usually associated with moderate thrombocytopenia, but severe thrombocytopenia may also occur (Likic and Kuzmanic, 2004). EBV binding to platelets activates TGF-β known to cause EBV reactivation and contributing to immunosuppression, while thrombocytopenia is a poor prognostic sign in EBV infections (Ahmad and Menezes, 1997; Kimura et al., 2003). Fall of platelets counts is also typical of other herpesevirus infections. Forghani and Schmidt (1983) have demonstrated that following *in vivo* inoculation with herpes simplex virus platelets contained much more virus than leukocytes. Recent data of Stokol et al. (2015) clearly indicate that a herpesvirus binds and activates platelets; the mechanisms of those interactions are unknown. Those findings suggest that such interactions may indeed cause pathologic sequelae such as inflammation, thrombosis, and the dissemination of viral infection.

The potential of phages to interfere with some viral infections is also highlighted by the data on hantavirus interactions with platelets which are facilitated by αIIbβ3 integrin; the role of that integrin is further supported by data indicating that its polymorphism may be a risk factor for hantavirus – induced disease while the intensity of levels of its expression on platelets correlate with disease severity.

Interestingly, hantavirus uses a non-RGD containing protein for its β3-integrin – dependent binding to platelets (Gavrilovskaya et al., 1999). Thus, it cannot be excluded that phages that do not contain a KGD motif could interact with cellular integrin receptors as well and thereby interfere with PV infectivity. Finally, a potential role of phages present in the human body should also be considered. Such phages are detectable in blood of patients on immunosuppressive therapy (Kowarsky et al., 2017). Moreover, phages are abundant when patients are on low immunosuppression but disappear when immunosuppression is high (De Vlaminck et al., 2013). This may suggest that immunosuppression may cause both immune deficiency and “phage deficiency” so phage-mediated protection may be lost. Thus, EBV-dependent syndromes in patients on immunosuppression may be induced by deficiency of immune system and endogenous phages.

## Conclusion

Drug repurposing (also referred to as drug repositioning) is a strategy currently attracting much attention aiming to apply existing medications for new indications, including those with entirely different profiles to that for which a drug had been developed. This approach is gaining popularity and – importantly – has been successful through observational studies and serendipity. For example, a well-known antidiabetic drug metformin is now being tested in >100 clinical trials as a potential anticancer agent and its other possible applications are on the horizon (Hernandez et al., 2017; see also Frontiers’ Research Topic: Metformin: Beyond Diabetes). It is quite likely that phage therapy may follow a similar pathway from treating bacterial infections to other medical applications. The advancement of knowledge about phages leads to gradual transformation of our understanding of their role from solely bacterial viruses toward a broader concept of phages as “guests protecting our health” (Guglielmi, 2017). Data are available to suggest that this emerging concept of phage-mediated protection may apply to possible phage interactions with integrins responsible for herpesvirus infections – specifically EBV. Moreover, the data indicating the existence of common mechanisms activating viral membrane fusion strongly suggest that it is possible to develop agents for therapeutic targeting of different herpesviruses (Sathiyamoorthy et al., 2017a). Phage competition with herpesviral binding of epithelial cells and B cells can contribute to prevention of those viral infections as well as prevention of reactivation of their lytic cycle. Furthermore, by blocking αvβ6 integrins phages could inhibit EBV-dependent and EBV-unrelated tumorigenesis. Hopefully, further studies could enable the translation of those findings to novel therapeutic means so urgently needed in viral infections.

## Author Contributions

AG drafted the main part of the manuscript. RM, EJ-M, BW-D, NB, and JB contributed parts of the manuscript. All authors approved the manuscript.

## Conflict of Interest Statement

AG, RM, BW-D, and JB are co-inventors of patents owned by the Institute and covering phage preparations. The other authors declare that the research was conducted in the absence of any commercial or financial relationships that could be construed as a potential conflict of interest.
